# Novel target and treatment agents for natural killer/T-cell lymphoma

**DOI:** 10.1186/s13045-023-01483-9

**Published:** 2023-07-22

**Authors:** Xiao-Peng Tian, Yi Cao, Jun Cai, Yu-Chen Zhang, Qi-Hua Zou, Jin-Ni Wang, Yu Fang, Jia-Hui Wang, Song-Bin Guo, Qing-Qing Cai

**Affiliations:** 1 Department of Medical Oncology, State Key Laboratory of Oncology in South China, Sun Yat-Sen University Cancer Center, No. 651, Dongfeng Road East, Guangzhou, 510060 People’s Republic of China; 2 State Key Laboratory of Oncology in South China, Collaborative Innovation Center of Cancer Medicine, Sun Yat-Sen University Cancer Center, Guangzhou, People’s Republic of China

**Keywords:** Natural killer/T-cell lymphoma, Targeted therapy, Immunotherapy, Novel agents

## Abstract

The rapidly increasing use of high-throughput screening had produced a plethora of expanding knowledge on the molecular basis of natural killer/T-cell lymphoma (NKTCL), which in turn has revolutionized the treatment. Specifically, the use of asparaginase-containing regimens has led to substantial improvement in survival outcomes in NKTCL patients. Novel treatment strategies that are currently under development include cell-surface-targeted antibodies, immune checkpoint inhibitors, Epstein-Barr virus targeted cytotoxic T lymphocyte, immunomodulatory agents, chimeric antigen receptor T cells, signaling pathway inhibitors and epigenetic targeted agents. In almost all cases, initial clinical studies of newly developed treatment are conducted in patients relapsed, and refractory NKTCL due to very limited treatment options. This review summarizes the results of these novel treatments for NKTCL and discusses their potential for likely use in NKTCL in a wider setting in the future.

## Background

Natural killer/T-cell lymphoma (NKTCL) is a rare and highly aggressive subtype of non-Hodgkin lymphoma (NHL) strongly associated with Epstein-Barr virus (EBV) infection and characterized by extranodal involvement [1, 2]. NKTCL cells express high level of P-glycoprotein and are thus resistant to anthracycline-containing therapies, such as the CHOP (cyclophosphamide, doxorubicin, vincristine, and prednisone) regimen [3]. Based on improved survival outcomes in NKTCL patients, therapeutic regimens based on L-asparaginase are now recommended by the NCCN guidelines [4]. These regimens include SMILE (dexamethasone, methotrexate, ifosfamide, L-asparaginase, and etoposide) regimen [5], modified SMILE (use of pegaspargase instead of L-asparaginase) [6], P-GemOx (pegaspargase, gemcitabine, and oxaliplatin) [7] and DDGP (dexamethasone, cisplatin, gemcitabine, and pegaspargase) [8, 9]. Despite these advances, survival outcome in patients with relapsed or refractory (r/r) disease remains poor. In patients who relapsed after initial non-anthracycline-based treatment, the median overall survival is only 6 months [10].

Genetic testing, particularly high-throughput sequencing, has radically changed the landscape of treatment of malignant tumors, including NKTCL [11, 12]. The current review summarizes the key molecular hallmarks of NKTCL and the corresponding targeted therapies (e.g., immune checkpoint inhibitors, cell surface-targeted agents, epigenetic targeted agents, signaling pathway inhibitors), mostly tested in patients with r/r NKTCL. This review also provides a perspective on future development.

### Cell-surface-targeted antibodies

**Fig. 1:**
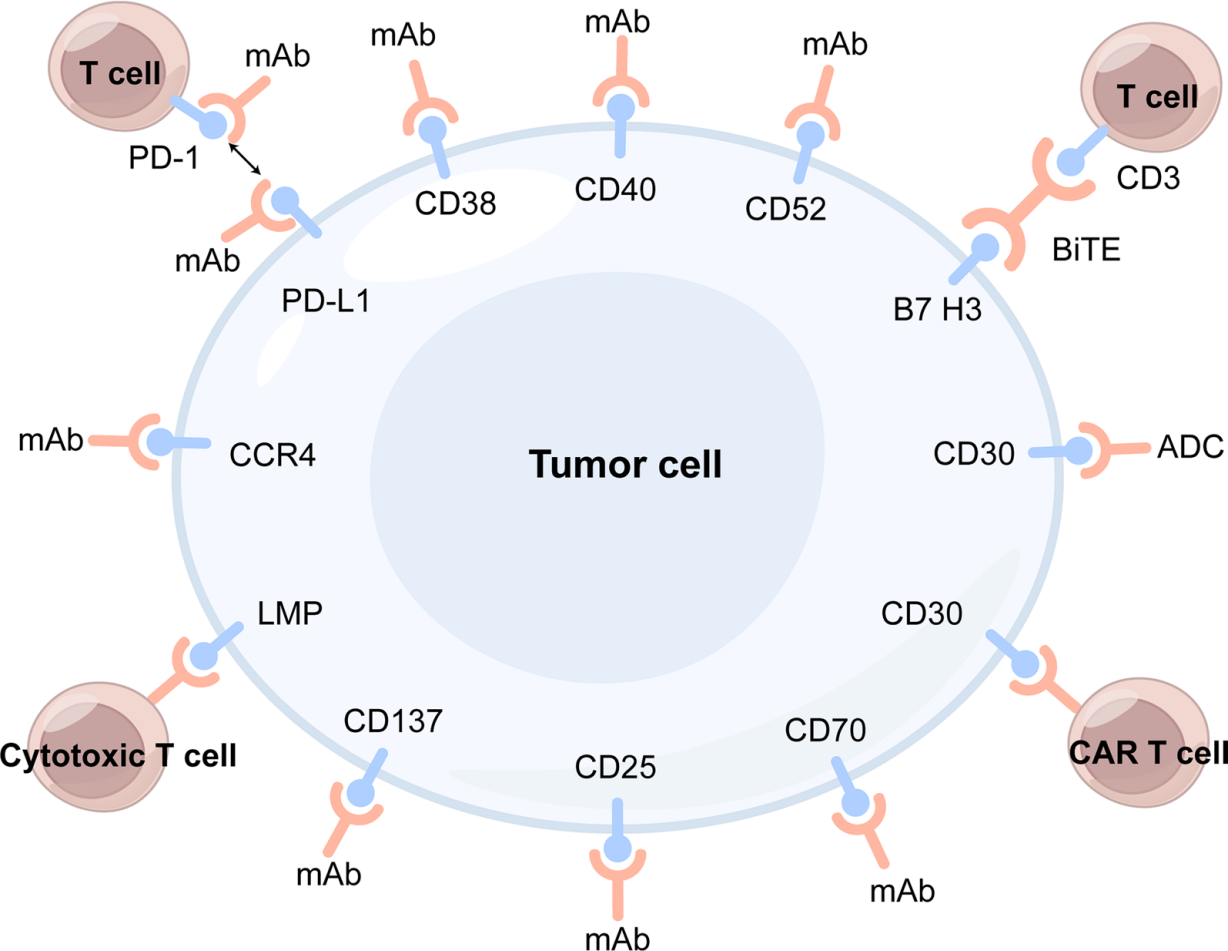
Cell-surface antigens as potential therapeutic targets for NKTCL. mAb: monoclonal antibody, ADC: antibody–drug conjugate, BiTE: bispecific T cell antigen, CAR: chimeric antigen receptor, LMP: latent membrane protein, PD-1: programmed cell death protein 1, PD-L1: programmed cell death ligand 1

A variety of cell surface antigens have been used to develop targeted therapy for NKTCL (Fig. 1). Monoclonal antibodies (mAbs) against cell surface antigens and their conjugated forms, such as antibody–drug conjugates (ADCs) and bispecific T-cell engagers (BiTEs), under development for NKTCL treatment are listed in Table 1.

**Table 1:** Summary of cell-surface molecules for targeted therapy and ongoing clinical trials in NKTCL patients

Agent		Target	Trial ID	Patient number (evaluable /estimate)	Study phase	Combined agents	Indication	Results for NKTCL	References
Cell-surface-targeted antibodies	Daratumumab	CD38	NCT02927925	32	2	/	r/r NKTCL	ORR:25%, CR:0% 4-m PFS:13% 6-m OS:42.9%	[16]
	Isatuximab	CD38	NCT04763616	37	2	Cemiplimab	r/r NKTCL	/	/
	Basiliximab	CD25	NCT04337593	30	2	Pegaspargase	r/r NKTCL	/	/
	Alemtuzumab	CD52	NCT00069238	31	2	EPOCH	Untreated T and NK-cell lymphoma	/	/
	Brentuximab vedotin	CD30	NCT02280785	33 (7 NKTCL)	2	/	r/r CD30-expressing NHL	ORR:29%	[22]
	Brentuximab vedotin	CD30	NCT03246750	36	1/2	MAD	Newly diagnosed ENKTL	/	/
CAR-T Therapy	CD30.CAR-T	CD30	NCT04526834	21	1		r/r CD30 positive NHL	/	/
	CD30.CAR-T	CD30	NCT03049449	26	1	Cyclophosphamide Fludarabine	CD30 expressing lymphomas	/	/
	CD30.CAR-EBVSTs	CD30	NCT04288726	18	1		r/r CD30 positive NHL	/	/
EBV targeted CTL	Baltaleucel-T	EBV antigens	NCT01948180	15	2		Advanced ENKTL	Salvage cohort: ORR:50% CR:30%	[73]
	VT-EBV-N	LMP	NCT03671850	48	2		EBV positive NKTCL		

*NKTCL*: natural killer/T-cell lymphoma, *ORR*: objective response rate, *CR*: complete remission, *OS:* overall survival, *PFS*: progression-free survival, *EPOCH*: etoposide, prednisone, vincristine, cyclophosphamide and doxorubicin, *NHL*: non-Hodgkin lymphoma, *MAD*: methotrexate, L-asparaginase, and dexamethasone, *ENKTL*: extranodal natural killer/T- cell lymphoma, *CAR*: chimeric antigen receptor, *CTL*: cytotoxic T lymphocyte, *EBV*: Epstein-Barr virus, *LMP*: latent membrane protein

### CD38-targeted mAbs

CD38 is a glycoprotein primarily expressed on the surface of T cells, NK cells, B cells, and other immune cells. CD38 functions as a lymphocyte receptor and transducer of signals to regulate the proliferation and differentiation of these cells [13]. A study of 94 patients with NKTCL found CD38 expression on NKTCL cells in majority of the cases and very high CD38 expression in half of the patients [14]. Daratumumab is an anti-CD38 mAb approved by the US FDA for use in patients with multiple myeloma (MM). The action of daratumumab is mediated by various Fc-dependent immune mechanisms, including antibody dependent cytotoxicity (ADCC), antibody dependent phagocytosis (ADCP), and complement dependent cytotoxicity (CDC) [15]. However, a phase 2 single-arm trial of 32 patients with r/r NKTCL reported limited efficacy: at a dose of 16 mg/kg, daratumumab monotherapy demonstrated 25% objective response rate (ORR), no complete remission (CR), 13% 4-month progression-free survival (PFS) rate and 43% 6-month overall survival (OS) rate [16]. Efforts are ongoing in developing anti-CD38 mAb (e.g., isatuximab) in combination with immunotherapy (e.g., cemiplimab, a programmed cell death protein 1 (PD-1) inhibitor) for NKTCL (NCT04763616) since CD38 has been shown to attenuate immune response to immune checkpoint therapy [17].

### CD30-targeted ADCs

CD30 is expressed on activated lymphocytes and can mediate multiple signaling pathways to modulate cell growth, proliferation and apoptosis [18]. CD30 is expressed in 40–75% of NKTCL patients [19–21]. Brentuximab vedotin (BV) is an ADC that combines an anti-CD30 mAb with monomethyl auristatin E, a microtubule-targeting cytotoxic agent, and has been studied in a variety of NHLs and demonstrated satisfactory efficacy and safety [22]. The ECHELON-2 compared a BV-CHP regimen (BV, cyclophosphamide, doxorubicin, and prednisone) versus CHOP regimen in patients with peripheral T-cell lymphoma (PTCL), and reported improved long-term patient survival with BV-CHP [23], facilitating the exploration of BV in NKTCL treatment. Complete remission after BV monotherapy or in combination with bendamustine in patients with refractory NKTCL has been reported in scattered case series [24, 25]. A phase 2 single-arm trial evaluated BV monotherapy in r/r NHL patients with high CD30 expression; in the 7 NKTCL patients, CR and partial remission (PR) was reported in 1 patient each [22]. One trial (NCT03246750) is ongoing to examine the efficacy of BV in combination with methotrexate, L-asparaginase, and dexamethasone (MAD) in patients with newly diagnosed NKTCL.

### CD52-targeted mAbs

CD52, a cell surface marker in mature lymphocytes, is expressed in 25% to 47% of NKTCL patients [26, 27]. The anti-CD52 mAb alemtuzumab has demonstrated encouraging efficacy and acceptable safety as monotherapy in patients with T cell lymphomas and other malignant hematopoietic diseases [28]. A trial of 116 patients with PTCL compared alemtuzumab in combination with CHOP versus CHOP regimen alone, and found higher CR rate with the combination regimen (60%) versus CHOP alone (43%) [29]. A trial of alemtuzumab plus EPOCH (etoposide, prednisone, vincristine, cyclophosphamide, and doxorubicin) regimen (NCT00069238) is currently ongoing in patients with untreated T and NK-cell lymphomas that include a subpopulation of NKTCL patients.

### CD25-targeted mAbs

CD25 is the alpha chain of interleukin-2 receptor (IL-2Rα) that increases the affinity of IL-2R complex to IL-2 by combining to the β and γ chains of IL-2R (IL-2R β and IL-2Rγ). The activation of IL-2R promotes cell proliferation and immune response [30]. In comparison to healthy volunteers, NKTCL patients had significantly higher serum CD-25 [31]. Elevated serum CD25 has been associated with poor response to chemotherapy and survival in NKTCL patients [31]. On the basis of PR after treatment with an anti-CD25 mAb basiliximab, plus pegaspargase in a patient with relapsed NKTCL [32], a phase 2 clinical trial of basiliximab plus pegaspargase is currently ongoing in NKTCL patients (NCT04337593).

### C-C chemokine receptor (CCR4)-targeted mAbs

Chemokines are implicated in hematologic malignancies, including progression, metastasis, and angiogenesis [33]. NKTCL patients have elevated serum chemokine (CC motif) ligand (CCL) 17 and CCL22 as well as expression of their receptor CCR4 in tumor tissues [34]. The anti-CCR4 mAb mogamulizumab has been shown to enhance the ADCC activity of NK cells against NKTCL cell lines [35]. In a phase 3 randomized trial of 372 patients with cutaneous T-cell lymphoma, the ORR was 28% in the mogamulizumab versus 5% in the vorinostat group [36]. Currently, there is no clinical trials of anti-CCR4 mAbs in NKTCL patients.

### CD40-targeted mAbs

CD40 is a member of the tumor necrosis factor receptor (TNFR) superfamily and is broadly expressed on the surface of both immune and non-immune cells [37]. CD40 is expressed in EBV-infected NKTCL cell lines, and CD40-CD40 ligand (CD40L) signaling has been shown to protect EBV-infected T/NK cells from apoptosis [38]. Dacetuzumab is a humanized immunoglobulin G1 mAb against CD40 developed initially for diffuse large B-cell lymphoma (DLBCL). A phase 2b trial in patients with relapsed DLBCL, however, failed to show improved survival with dacetuzumab [39]. Currently, there is no trial of CD40-targeted agent in NKTCL patients.

### B7-H3-targeted BiTEs

B7-H3 (also known as CD276) is a member of the B7 ligand family, and functions as a protumorigenic factor to inhibit immune response in malignant tissues [40]. Zheng et al. [41] discovered that B7-H3 is highly expressed in NKTCL cell lines, and constructed a BiTE antibody that connects B7-H3 to the CD3 chain of T cell receptor (TCR) complex to achieve specific T cell cytotoxicity against NKTCL cells [42]. Preclinical studies demonstrated encouraging results in cultured NKTCL cells as well as a mouse model of NKTCL [41]. Currently, there is no trial of B7-H3 targeted therapy in NKTCL patients.

### Other potential targets

A previous study confirmed the high expression of CD70 in SNK6 and SNK8 cell lines as well as tumor cells from NKTCL patients [43]. In that study, anti-CD70 mAb induced complement-dependent killing of SNK-6 cells. CD137 is also highly expressed in NKTCL cell lines, likely due to the induction by latent membrane protein 1 (LMP1) encoded by EBV [44]. CD137 deficiency hampers T cell proliferation [45, 46]. The studies of CD70 and CD137 are in the very early stage of preclinical development.

### Immune checkpoint inhibitors (ICIs)

Immune checkpoints, e.g., PD-1 and programmed cell death ligand 1 (PD-L1), are critical in the development and maintenance of immune tolerance in tumor microenvironment [47]. PD-L1 is expressed in 39% to 100% of NKTCL patients [48–51]. Previous studies demonstrated a close association between EBV infection and PD-L1 expression in various malignancies [52, 53]. PD-L1 expression in NKTCL is increased by EBV-driven LMP1 through the nuclear factor κB (NF-κB) signaling pathway [54]. Many ICIs, including the PD-1 mAbs pembrolizumab, sintilimab, tislelizumab, and toripalimab, the PD-L1 mAbs sugemalimab and avelumab, and the dual-targeting anti-PD1/PD-L1 antibody IBI318, have been investigated for NKTCL (Table 2).

**Table 2:** Summary of immune checkpoint inhibitors and ongoing clinical trials in NKTCL patients

Agent	Target	Trial ID	Patient number (evaluable /estimate)	Study phase	Combined agents	Indication	Results for NKTCL	References
Avelumab	PD-L1	NCT03439501	21	2	/	r/r NKTCL	ORR: 38% CR:24%	[60]
Sugemalimab (CS1001)	PD-L1	NCT05700448	150	3	P-GemOx	r/r NKTCL	/	/
Sugemalimab (CS1001)	PD-L1	NCT03595657	80	2	/	r/r ENKTL	ORR:46.2%, CR:30.4% 1y OS:68.6% 2y OS:54.6%	[61, 62]
IMC-001	PD-L1	NCT04414163	20	2	/	r/r NKTCL	/	/
Camrelizumab (SHR-1210)	PD-1	NCT03363555	97	2	/	r/r NKTCL	/	/
Toripalimab	PD-1	NCT04365036	207	3	P-GemOx	Newly diagnosed early stage NKTCL	/	/
Sintilimab	PD-1	NCT03228836	28	2	/	r/r NKTCL	ORR: 75.0% 1y OS:82.1% 2y OS: 78.6%	[58]
Sintilimab	PD-1	NCT04279379	20	2	Decitabine	r/r or advanced NKTCL	/	/
Sintilimab	PD-1	NCT04127227	63	2	P-GemOx	Newly diagnosed advanced ENKTL	ORR:100% CR:87.5% 1y OS:100% 1y PFS:95%	[126]
Sintilimab	PD-1	NCT05008666	37	2	Chidamide Azacitidine L-DEP	ENKTL-HLH	/	/
Sintilimab	PD-1	NCT04676789	30	2	Pegaspargase	Limited stage NKTCL	/	/
Sintilimab	PD-1	NCT03936452	55	2	Pegaspargase Anlotinib	Untreated, limited stage NKTCL	ORR:87.8% CR:87.8% 2y PFS:87.6% 2y OS:97.9%	[127]
Tislelizumab	PD-1	NCT05477264	38	2	/	Newly diagnosed NKTCL	/	/
Immune Tislelizumab	PD-1	NCT05254899	54	2	P-GemOx	High-risk early stage ENKTL	/	/
Tislelizumab	PD-1	NCT05464433	46	1/2	Mitoxantrone hydrochloride liposome	r/r NKTCL	/	/
Tislelizumab	PD-1	NCT05058755	62	NA	/	r/r NKTCL	/	/
Tislelizumab	PD-1	NCT04038411	50	2	Chidamide, Lenalidomide, Etoposide	r/r NKTCL	CR:50.0% 1y PFS:86.8%	[76]
Tislelizumab	PD-1	NCT03493451	77 (22 NKTCL)	2	/	r/r mature T- and NK-cell neoplasms	ORR 31.8% CR 18.2%	[128]
Pembrolizumab	PD-1	NCT04417166	30	2	/	Untreated, limited stage NKTCL	/	/
Pembrolizumab	PD-1	NCT03728972	19	2	/	Untreated, early-stage ENKTL	/	/
Pembrolizumab	PD-1	NCT03107962	20	2	/	r/r NKTCL	/	/
IBI318	PD-1 and PD-L1 bispecific	NCT04602065	129	1/2	/	r/r NKTCL	/	/

*PD-1*: programmed cell death protein 1, *PD-L1*: programmed cell death ligand 1, *NKTCL*: natural killer/T-cell lymphoma, *ORR*: objective response rate, *CR*: complete remission, *PR*: partial remission, *OS:* overall survival, *PFS*: progression-free survival, *P-GemOx*: pegaspargase, gemcitabine, and oxaliplatin, *ENKTL*: extranodal natural killer/T-cell lymphoma, *L-DEP*: L-asparaginase, doxorubicin liposome, etoposide and methylprednisolone

In a retrospective study of 7 r/r NKTCL patients, pembrolizumab monotherapy at a dose of 2 mg/kg every 3 weeks showed 100% ORR without significant toxicities; notably, 5 patients (71%) achieved CR, and all remained in CR after a median follow-up of 6 months [55]. In another retrospective study in 7 r/r NKTCL patients, pembrolizumab monotherapy at a dose of 100 mg every 3 weeks showed 57% ORR [56]. Two trials (NCT04417166, NCT03728972) are ongoing to examine pembrolizumab monotherapy in patients with untreated early-stage NKTCL. Nivolumab was evaluated in a study that included 3 patients who had failed previous L-asparaginase-based regimens [57]. In this study, all 3 patients showed initial response, but only 1 patient remained in CR and the remaining 2 patients died from infections. In the phase 2 single-arm ORIENT-4 trial [58], monotherapy with the anti-PD-1 mAb sintilimab demonstrated 75.0% ORR and 78.6% 2-year OS in 28 patients with r/r NKTCL. Several other ICIs, including toripalimab, camrelizumab, tislelizumab and IBI318, are being investigated in ongoing clinical trials (Table 2). In a study of 9 patients with advanced NKTCL [59], PD-1 inhibitors in combination with P-GemOx chemotherapy demonstrated 88.9% ORR, 77.8% CR rate, 66.7% 1-year PFS rate and 100.0% 1-year OS rate. A phase 2 trial (NCT04127227) is ongoing to examine PD-1 inhibitors in combination P-GemOx chemotherapy in patients with advanced NKTCL (Table 2).

In a phase 2 trial of 21 patients with r/r NKTCL, monotherapy with the anti-PD-L1 mAb avelumab demonstrated 38% ORR and 24% CR rate [60]. However, the responders in this trial had relatively long remission, with the maximum that exceeded 25 months [60]. This study also demonstrated a positive correlation between PD-L1 expression and treatment response, suggesting that evaluating the level of PD-L1 expression could potentially help identify patients who are more likely to benefit from PD-L1 inhibitors. In a single-arm phase 2 trial in 80 patients with r/r NKTCL, the anti-PD-L1 mAb sugemalimab demonstrated 46.2% ORR, 30.4% CR rate, 68.6% 1-year OS rate and 54.6% 2-year OS rate [61, 62]. In addition to EBV-driven LMP1, PD-L1 can also be increased via the signal transducer and activator of transcription 3 (STAT3) [63]. Given these results, it is possible to combine PD-L1 inhibitors with other therapies such as STAT3 inhibitors and EBV-targeted cytotoxic T lymphocytes (CTL). Other PD-L1 inhibitors (e.g., IMC-001) are summarized in Table 2.

Many other immune checkpoint molecules, including transforming growth factor-β1 (TGF-β1), cytotoxic T lymphocyte-associated antigen 4 (CTLA-4), T-cell immunoglobulin-3 (TIM-3), T-cell immunoglobulin and ITIM domain (TIGIT), B/T lymphocyte attenuator (BTLA), and lymphocyte-activation gene 3 (LAG-3), are upregulated in NKTCL patients [64, 65]. However, there are currently no targeted therapies based on these molecules [64, 65].

### EBV targeted cellular therapy

EBV is a one of the most ubiquitous viruses that infect human beings, with an estimated prevalence of 90% in the world population [66]. The latency II pattern EBV antigens, including LMP1, LMP2 and EBV nuclear antigen 1 (EBNA1), are widely expressed in EBV-positive tumor cells [67, 68], and have been implicated in the survival and proliferation of NKTCL cells [69]. LMP1 promotes the survival, proliferation, invasion and migration of NKTCL cells through the NF-κB pathway [70]. In a phase 1 single-arm trial of 52 patients with EBV-associated lymphomas, treatment with CTLs that target LMP2 or LMP1/2 resulted in long-lasting responses without significant toxicity [71]. This study included 11 patients with NKTCL, among whom 6 had active disease and 5 were in remission but at high risk for relapse. Four out of the 6 patients with active disease achieved CR and 3 of them remained in CR for at least four years. All 5 patients in remission remained in remission for at least 2 years after CTLs infusion. In a study of 10 NKTCL patients (8 with localized disease and 2 with advanced disease) who received autologous LMP-specific CTLs after achieving CR with induction therapy, the 4-year OS and PFS rate were 100% and 90%, respectively [72]. While the results of this study are impressive, further research is necessary since most patients had localized-stage NKTCL. Kim et al. [73] conducted a phase 2 trial to examine autologous EBV-specific T cells (baltaleucel-T) in 54 patients with advanced, relapsed NKTCL. The attempt to expand baltaleucel-T cells failed in 39 out of the 54 patients. The remaining 15 patients received a median of 4 doses of baltaleucel-T. In the 10 patients with active disease, the therapy achieved 50% ORR and 30% CR rate. In the remaining 5 patients with no measurable disease at the baseline, 2 remained in remission during the follow-up of 5 months.

Overall, these studies showed that EBV targeted cellular therapies could induce sustained treatment response in NKTCL patients. However, there is a major need to optimize the CTL expansion protocol.

### Immunomodulatory agents

A randomized trial in NKTCL patients reported moderate efficacy of immunomodulatory agent thalidomide in combination with conventional chemotherapy: 8 out the 11 patients achieved CR, and one achieved PR [74]. Successful treatment of relapsed NKTCL with lenalidomide was described in a case report. This patient experienced relapse after hematopoietic stem cell transplantation (HSCT), and achieved CR with lenalidomide monotherapy [75]. Further studies with larger sample sizes are needed to verify these preliminary findings. In a phase 2 single-arm trial of in 20 r/r NKTCL patients, lenalidomide plus tislelizumab, chidamide and etoposide demonstrated 50.0% CR and 86.8% 1-year PFS rate [76]. A phase 3 trial (NCT02085655) is ongoing to compare P-GemOx plus thalidomide with AspaMetDex (pegaspargase, methotrexate, dexamethasone) in previously untreated or r/r NKTCL (Table 3).

**Table 3:** Summary of epigenetic targeted therapy, and immunomodulatory agents and ongoing clinical trials in NKTCL patients

Agent		Target	Trial ID	Number of estimated enrollment	Study phase	Combined agents	Indication	Results for NKTCL	References
Immuno-modulatory agent	Thalidomide	/	NCT02085655	264	3	P-Gemox or methotrexate, dexamethasone	Previously untreated or r/r NKTCL	/	/
Epigenetic targeted agents	Chidamide	HDACi	NCT03820596	37	1/2	Sintilimab	r/r ENKTL	CR:59.5% PR:48.6% 1.5yPFS:52.5% 1.5y OS:76.2%	[120]
	Chidamide	HDACi	NCT03630731	32	2	/	Chemotherapy responded stage IV or r/r NKTCL	/	/
	Chidamide	HDACi	NCT04414969	35	2	Anti PD-1 antibody, Pegaspargase	Stage IE and IIE ENKTL	/	/
	Chidamide	HDACi	NCT04994210	30	2	Sintilimab	Newly diagnosed ENKTL	/	/
	Chidamide	HDACi	NCT02878278	24	2	/	r/r NKTCL	CR: 33%	[118]
	Vorinostat	HDACi	NCT00336063	18	1	Azacitidine	Nasal NKTCL	/	/
	Romidepsin	HDACi	NCT01913119	16	1	/	r/r NKTCL	/	/

*NKTCL*: natural killer/T-cell lymphoma, *HDACi*: histone deacetylase inhibitor, *CR*: complete remission, *ENKTL*: extranodal natural killer/T-cell lymphoma, *HLH*: hemophagocytic lymphohistiocytosis, *L-DEP*: L-asparaginase, doxorubicin liposome, etoposide and methylprednisolone, *P-GemOx*: pegaspargase, gemcitabine, and oxaliplatin

### Chimeric antigen receptor T-cell (CART) therapy

CART therapy has been successfully used in the treatment of aggressive B cell lymphomas [77, 78]. However, the use of CART therapy for NKTCL is limited. In a mouse model for NKTCL, CAR-T cells that target B7-H3 demonstrated robust cytotoxicity against NKTCL cells [79]. Several trials of CD30 CART therapy are currently ongoing in patients with CD30-positive NHL (NCT04526834, NCT03049449, NCT04288726). Since NKTCL is closely associated with EBV infection [1], LMP1 may represent another target for CART therapy for NKTCL.

### Signaling pathway inhibitors

**Fig. 2:**
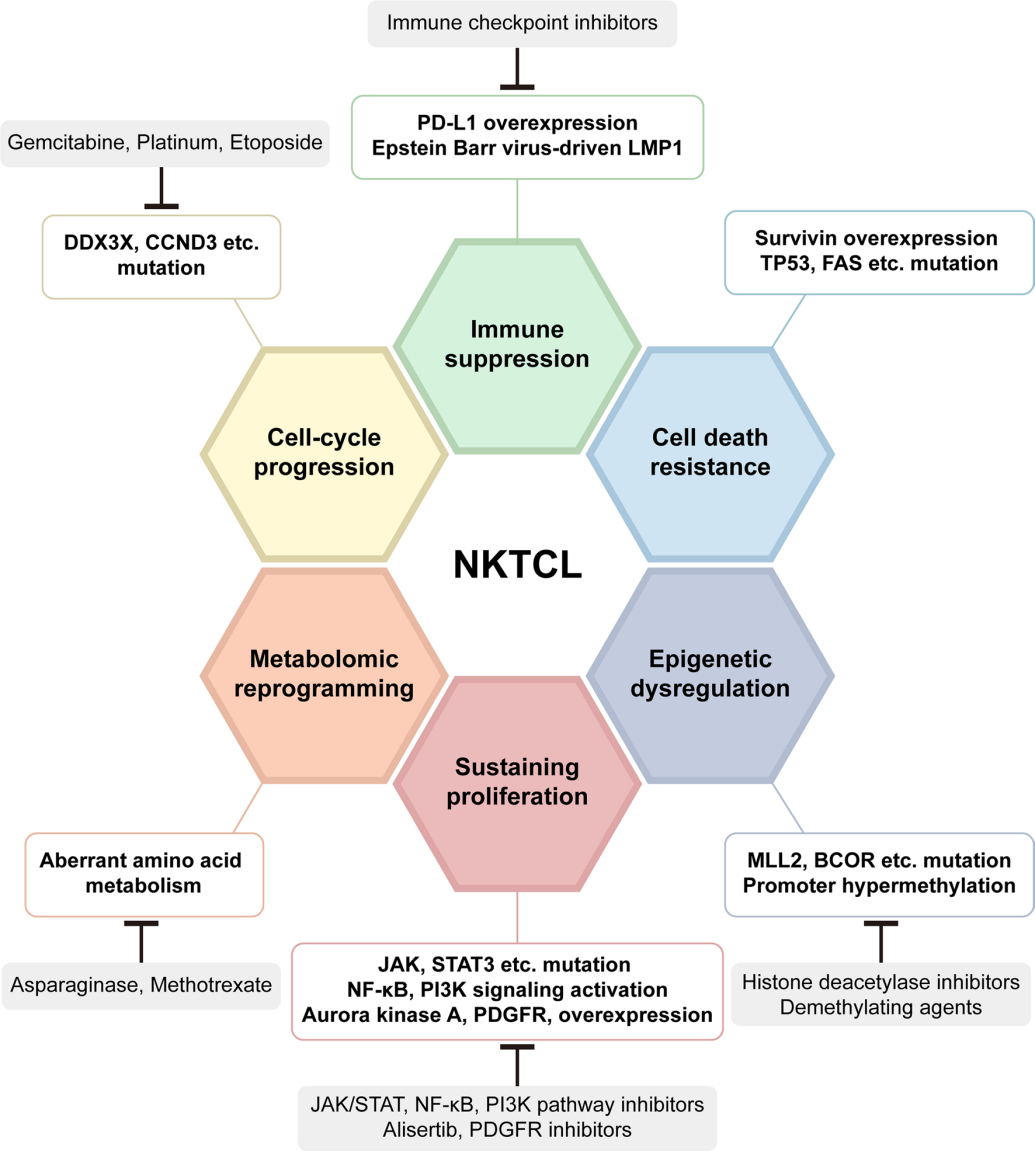
An overview of the six hallmark characteristics in the pathogenesis of NKTCL and targeted therapies. NKTCL: natural killer/T-cell lymphoma, PD-L1: programmed cell death ligand 1, MLL2: mixed lineage leukemia 2, BCOR: BCL-6 corepressor, JAK/STAT: Janus-associated kinase/signal transducer and activator of transcription, PDGFR: platelet-derived growth factor receptor, PI3K: phosphatidylinositol 3-kinase, NF-κB: nuclear factor κB, LMP1: latent membrane protein 1

**Fig. 3:**
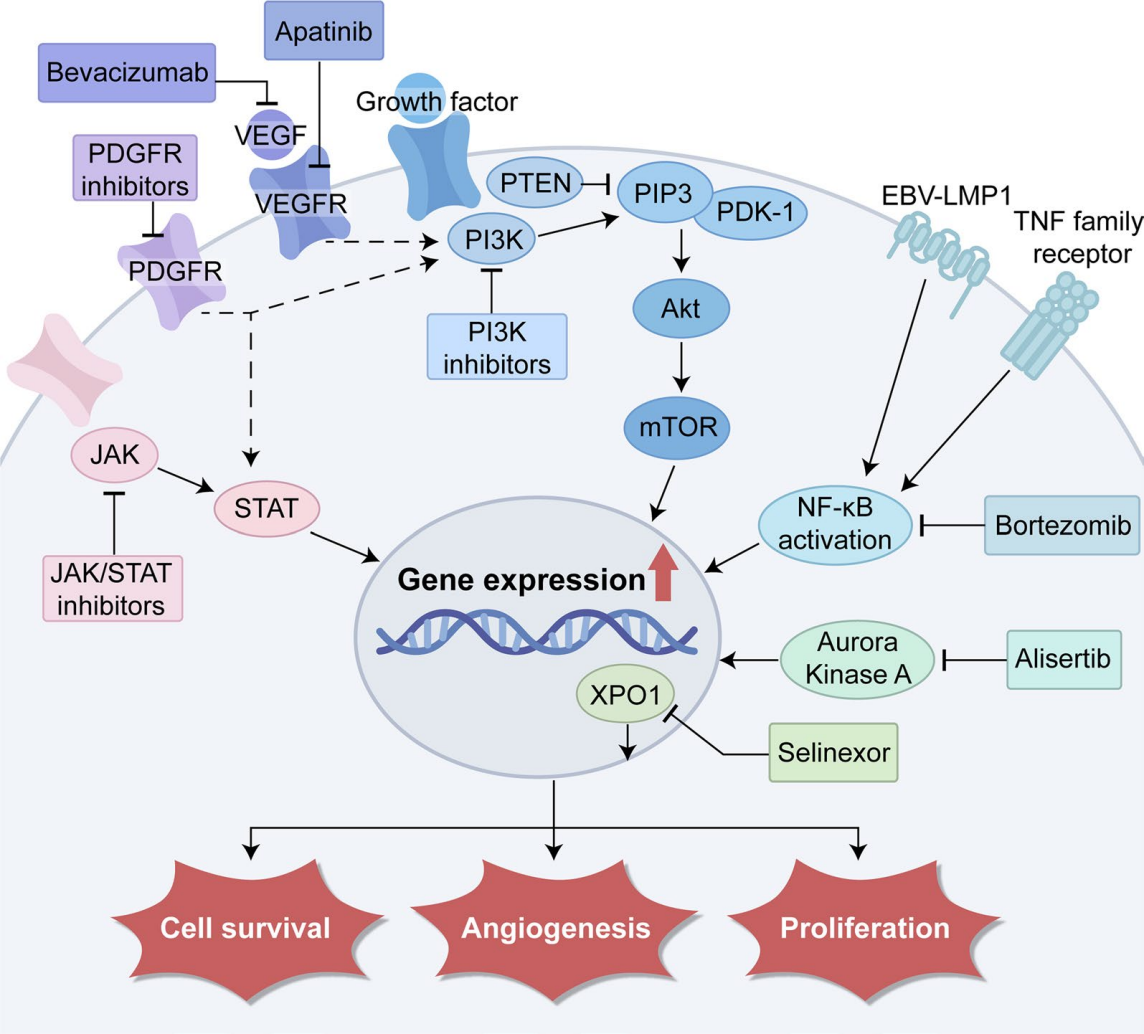
Novel agents targeting signaling pathways, including JAK/STAT, NF-κB, PDGFR, VEGF/VEGFR, PI3K/Akt/ mTOR, XPO1 and AURKA. PDGFR: platelet-derived growth factor receptor, VEGF: vascular endothelial growth factor, PI3K/Akt: phosphatidylinositol 3-kinase/protein kinase B, mTOR: mammalian target of rapamycin, XPO1: exportin-1, AURKA: Aurora kinase A

Genomic expression profiling (GEP) has unveiled a variety of mechanisms that underlie the pathogenesis of NKTCL [80], and holds the potential for developing individualized treatment strategies. Figure 2 is a schematic overview of the six main hallmark characteristics in the pathogenesis of NKTCL and the corresponding targeted therapies. Relevant signaling pathways include the Janus-associated kinase/signal transducer and activator of transcription (JAK/STAT), vascular endothelial growth factor (VEGF), platelet-derived growth factor receptor (PDGFR), phosphatidylinositol 3-kinase (PI3K)/ protein kinase B (Akt)/ mammalian target of rapamycin (mTOR) pathway and NF-κB pathways [11] (Fig. 3). In 2020, Xiong et al. [81] identified three molecular subtypes (TSIM, MB, and HEA) in NKTCL using an integrated approach combining whole-genome/exome sequencing, array-based copy number variation analysis, and RNA sequencing. In this study, activation of JAK/STAT and NF-κB pathway was involved in TSIM and HEA subtype, respectively, and these molecular subtypes were sensitive to different targeted treatments. In the following sections, we summarize the potential signaling pathway inhibitors against NKTCL (also in Table 4).

**Table 4:** Summary of signaling pathway inhibitors and ongoing clinical trials in NKTCL patients

Signaling pathway inhibitors	Target	Trial ID	Number of estimated enrollment	Study phase	Combined agents	Indication	Results for NKTCL	References
Bortezomib	NF-κB	NCT02808091	7	2	GIFOX	Newly diagnosis NKTCL	ORR:43%	[95]
Anlotinib	VEGFR	NCT04004572	37	2	Sintilimab Pegaspargase	Stage IV NKTCL	/	/
Apatinib	VEGFR2	NCT04366128	60	NA	Camrelizumab Pegaspargase	Stage IE/IIE ENKTL	/	/
Avastin (Bevacizumab)	VEGF	NCT01921790	30	2	GemAOD	Untreated NKTCL	/	/
Tofacitinib	JAK1/3	NCT03598959	20	2	Chidamide	r/r NKTCL	/	/
Ruxolitinib	JAK1/2	NCT02974647	82	2		r/r NKTCL	/	/
Selinexor (ATG-010)	XPO1	NCT04425070	97 (10 NKTCL)	1/2	GemOx or ICE or Tislelizumab	Peripheral T- and NK/T-cell lymphoma	ORR: 60% CR: 20%	[109]

*NKTCL*: natural killer/T-cell lymphoma, *GIFOX*: gemcitabine, ifosfamide and oxaliplatin, *ORR*: objective response rate*, CR*: complete remission, *GemAOD*: gemcitabine, oxaliplatin, pegaspargase and dexamethasone, *VEGFR*: vascular endothelial growth factor receptor, *VEGF*: vascular endothelial growth factor, *NA*: not available, *JAK*: Janus-associated kinase, *NF-κB*: nuclear factor κB, *ENKTL*: extranodal natural killer/T-cell lymphoma, *XOP1*: exportin-1, *GemOx*: gemcitabine and oxaliplatin, *ICE*: ifosfamide, carboplatin and etoposide

### JAK/STAT pathway inhibitors

Aberrant activation of JAK/STAT pathway is responsible for sustained proliferation of tumor cells under the stimulation by various cytokines [82, 83], and is a crucial factor in the pathogenesis of NKTCL [84]. In a study of 65 patients with NKTCL by Koo et al. [85], *JAK3* mutation was detected in 35.4% of the cases. In contrast, *STAT3* mutation is much less common (about 20%), but phosphorylated STAT3 (pSTAT3) is constitutively expressed in about 75% of NKTCL patients [86, 87]. These findings suggest that JAK/STAT pathway may be a potential therapeutic target in NKTCL. The pan-JAK inhibitor CP-690550 [85], the JAK1/3 inhibitor tofacitinib [87] and the selective STAT3 inhibitor WP1066 [86] could inhibit the proliferation and induce apoptosis in several NKTCL cell lines. *STAT3* mutations sensitize NKTCL cells to the STAT3 inhibitor static [87]. NKTCL cells with mutations in *JAK3* and *STAT3* are more susceptible than wild-type controls to treatment with tofacitinib and stattic, respectively [88]. Patients with *STAT3* mutation tend to have high expression of CD30 [21], suggesting that combining JAK/STATs inhibitors with an anti-CD30 antibody may be more effective. Two trials are currently ongoing to evaluate JAK inhibitors, tofacitinib in one trial (NCT03598959) and ruxolitinib in another (NCT02974647) in patients with r/r NKTCL. A phase 2 multinational global study evaluated the JAK1 selective inhibitor golidocitinib in r/r PTCL patients; in the 3 NKTCL patients, ORR was reported in 2 patients [89].

### NF-κB pathway inhibitors

NF-κB plays an important role in the proliferation and survival of immune cells [90]. Aberrant activation of the NF-κB pathway (e.g., elevated expression of *TNFRSF21* and *c-Rel*) has been recognized as a hallmark of NKTCL [91]. Both non-canonical pathway [92] and canonical pathway [93] have been implicated. Bortezomib, a proteasome inhibitor that indirectly inhibits the NF-κB pathway, has been shown to impair the viability and induce apoptosis of NKTCL cell lines [94]. In a phase 2 trial that enrolled only 7 NKTCL patients before termination due to slow recruitment [95], a combination of bortezomib and GIFOX (gemcitabine, ifosfamide, oxaliplatin) regimen demonstrated 43% ORR (CR in one patient). Bortezomib has been shown to trigger EBV into the lytic cycle from latency [96], suggesting the possibility of combination treatment with bortezomib and EBV targeted CTLs. Despite these findings, evidence for NF-κB targeted therapy for NKTCL is rather limited.

### VEGF/VEGFR inhibitors

GEP analysis revealed overexpression of angiogenesis-related genes in NKTCL cell lines, including *VEGF-A* and *KDR* (encoding VEGF-A and VEGF receptor 2 (VEGFR2), respectively) [91]. In a phase 2 trial of 39 patients with T cell lymphomas, the anti-VEGF mAb bevacizumab in combination with CHOP chemotherapy achieved 90% ORR [97]. Unfortunately, this trial did not include NKTCL patients. A phase 2 trial (NCT01921790) is ongoing to evaluate bevacizumab in combination with chemotherapy in NKTCL patients. VEGFR inhibitors apatinib and anlotinib are also being tested in several ongoing trials (NCT04004572, NCT04366128).

### PDGFR inhibitors

PDGFRα is a receptor tyrosine kinase that interacts with key proteins in both the JAK/STAT and PI3K/Akt signaling pathways [98]. GEP analysis revealed overexpression of *PDGFRα* and enhanced PDGFRα phosphorylation in NKTCL cell lines [91]. High PDGFRα expression has been associated with poor prognosis in NKTCL patients [99]. The PDGFR tyrosine kinase inhibitor imatinib has been shown to inhibit the viability of NKTCL cells and induce an arrest of cell cycle at G0/G1 stage [100]. However, few PDGFR pathway-related gene mutations have been reported in NKTCL cells so far, limiting the enthusiasm on PDGFR as a target for the treatment of NKTCL.

### PI3K/Akt/mTOR pathway inhibitors

The PI3K/Akt/mTOR pathway plays an important role in the regulation of cell proliferation and survival, and dysregulated PI3K/Akt/mTOR pathway is a hallmark of a variety of cancers [101]. A study showed high expression of several PI3K isoforms (PIK3α, PIK3β, PIK3γ, PIK3δ) in majority of NKTCL samples as well as an association between high PI3Kα expression with poor patient prognosis. This study also showed that copanlisib (a pan-class I inhibitor against PI3Kα and PIK3δ) could reduce the phosphorylation of Akt and inhibit the tumor growth both in vivo and in vitro [102]. In a study by Kawada et al., mTOR inhibitors (rapamycin and CCI-779) arrested NKTCL cells in the G1 phase and reduced cell viability [103], suggesting that mTOR inhibitor may also be a potential therapeutic option in NKTCL treatment. Overall, the PI3K/Akt/mTOR pathway represents promising target in developing new treatment of NKTCL, but no trials have been or are currently being conducted.

### Exportin-1 inhibitors

Exportin-1 (XPO1) facilitates the transport of various proteins and RNAs from the nucleus to the cytoplasm [104]. XPO1 has been reported to be overexpressed in NHLs and high expression of XPO1 has been associated with poor prognosis [105]. Selinexo, a selective XPO1 inhibitor, has been widely explored and demonstrated satisfactory efficacy in the treatment of DLBCL [106] and MM [107], but studies on NKTCL are limited. In a phase 1 trial of 10 patients with PTCL and 1 patient with NKTCL, selinexor in combination with DICE (dexamethasone, ifosfamide, carboplatin, and etoposide) regimen achieved 91% ORR and 82% CR rate, but 45% of patients discontinued the treatment due to the significant toxicities [108]. A phase 1b trial (NCT04425070) is currently ongoing to examine selinexor in combination with GemOx, ICE or tislelizumab in patients with PTCL and NKTCL. The latest results about the regimen of selinexor with GemOx are encouraging: 60% ORR and 20% CR in 10 NKTCL patients [109].

### Aurora kinase A inhibitors

Aurora kinase A (AURKA) plays a crucial role in the regulation of cell cycle, primarily during mitosis [110]. Overexpression of AURKA has been found in various hematological malignancies, including acute myeloid leukemia, MM and NHL [111]. A previous study observed high expression of AURKA in NKTCL cell lines as well as in NKTCL patients [112]. In this study, MK-8745 (a small-molecule AURKA inhibitor) significantly increased the apoptosis of NKTCL cells and induced the cell cycle arrest. However, there is no trial of AURKA targeted agents in the treatment of NKTCL patients.

### Epigenetic targeted agents

Epigenetic dysregulation has been described in a wide variety of solid and hematological malignancies [113, 114]. Mutations and aberrant expression patterns of BCL-6 corepressor (BCOR) and mixed lineage leukemia 2 (MLL2) have been implicated in NKTCL [115, 116]. Treatment approaches based on epigenetics for NKTCL are summarized in Table 3.

Histone deacetylase inhibitors (HDACi) produce multiple cytotoxic effects on cancer cells through histone acetylation of tumor suppressors [117]. Chidamide is a selective inhibitor of HDAC1, 2, 3 and 10 [118], and has been tested as monotherapy in a phase 2 trial in patients with r/r NKTCL (NCT02878278). In the most recent report of this trial [118], chidamide achieved 33% CR with a median follow-up of 3.7 months; all patients who achieved CR remained disease-free for > 6 months. In a non-randomized study of 37 patients with advanced NKTCL, the ORR was 40% in the 19 patients who received chidamide plus chemotherapy versus 15% in the remaining 18 who received chidamide monotherapy [119]. In a phase 1b/2 single-arm trial in r/r NKTCL patients, combination treatment with chidamide and sintilimab achieved 59.5% ORR, 48.6% CR, 52.5% 18-month PFS and 76.2% 18-month OS [120]. Several trials are currently being conducted to test chidamide in combination with other agents for NKTCL (NCT04994210, NCT05008666). In a trial of NKTCL patients, panobinostat, (an oral non-selective HDACi) in combination with bortezomib resulted in PR in one out of the two patients with r/r NKTCL [121]. Inhibition of histone deacetylase may trigger EBV reactivation and the use of romidepsin (another HDACi) has been reported to cause EBV reactivation [122, 123]. The risk of EBV reactivation when using HDACi therapy must be carefully considered and future strategy using anti-EBV drugs in combination with HDACi could be further explored. The ongoing trials evaluating HDACi in patients with NKTCL are summarized in Table 3.

Global promoter methylation analysis revealed hypermethylation of the promoters for a number of tumor suppressor genes, including *BCL2L11 (BIM), DAPK1*, and *TET2*, in NKTCL cell lines and patient samples; treatment with decitabine induced the re-expression of methylated genes [124], suggesting possible therapeutic action of demethylating agents.

## Conclusions

**Fig. 4:**
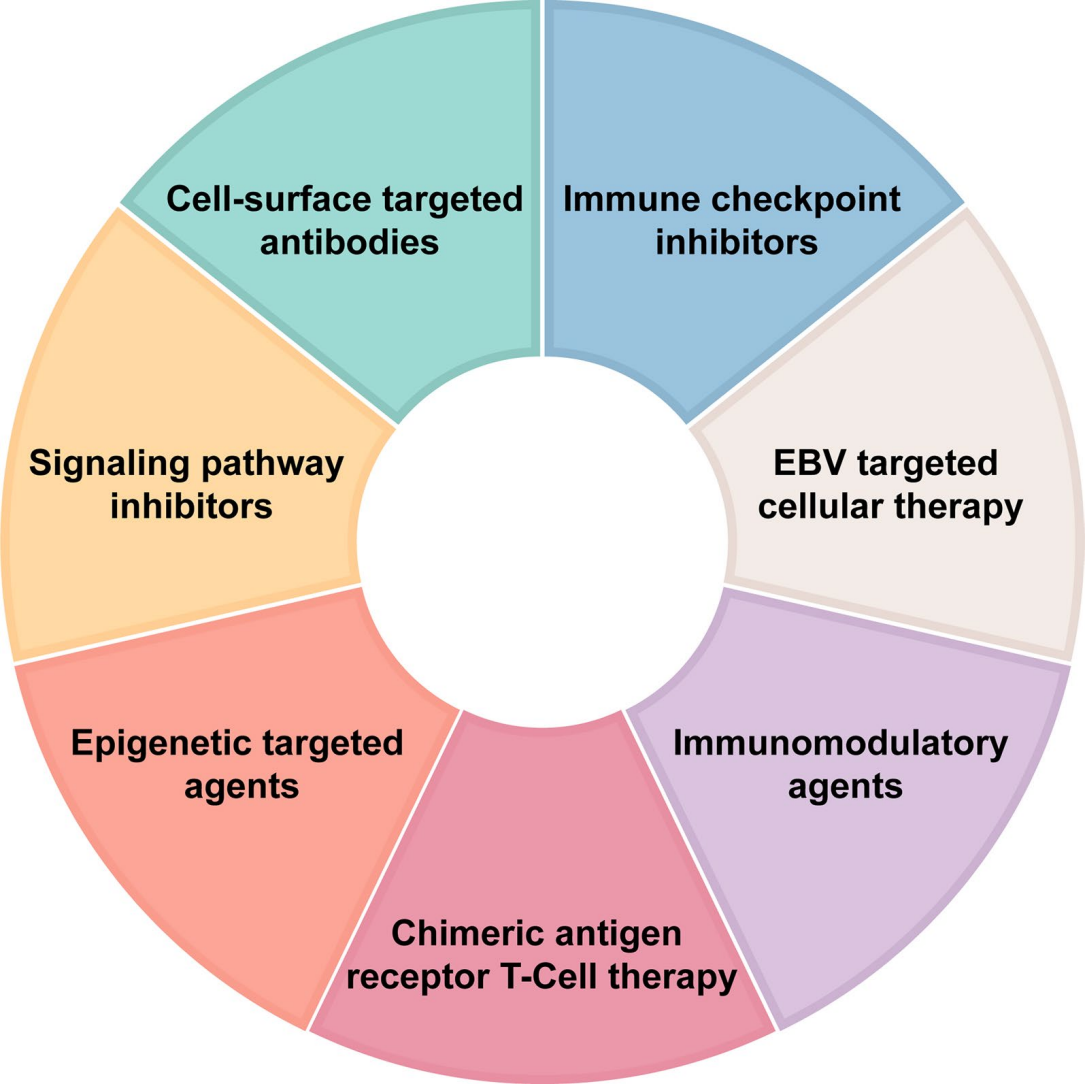
An overview of the seven main categories of novel agents for the treatment of NKTCL

Asparaginase-based chemotherapy has improved survival outcomes in patients with localized NKTCL. Advanced NKTCL, however, remains a major challenge, with disease progression within 5 years of diagnosis in over 70% of the patients [125]. A variety of novel agents have been developed for r/r NKTCL (summarize in Fig. 4). Among these novel treatments, immunotherapies (ICIs, cell-surface-targeted antibodies and EBV-specific CTL) have demonstrated promising results. Evidence of signaling pathway inhibitors and epigenetic targeted agents are currently limited. In our opinion, it is unlikely that signaling pathway inhibitors and epigenetic targeted agents could achieved satisfactory efficacy as monotherapy in r/r NKTCL. Combination strategies with ICIs, cell-surface-targeted antibodies and anthracycline-containing chemotherapy may help to enhance therapeutic efficacy in the management of r/r NKTCL and hold the potential for future development. Other important obstacles include limited number of patients available for clinical trials and distinct gene mutations in different patients. An individualized approach is thus required.

## Data Availability

The datasets supporting the conclusions of this study are included in the figures and tables.

## Abbreviations

NKTCL: Natural killer/T-cell lymphoma
EBV: Epstein-Barr virus
r/r: Relapsed and refractory
NHL: Non-Hodgkin lymphoma
CHOP: Cyclophosphamide, doxorubicin, vincristine, and prednisone
SMILE: Dexamethasone, methotrexate, ifosfamide, l-asparaginase, and etoposide
P-GemOx: Pegaspargase, gemcitabine, and oxaliplatin
DDGP: Dexamethasone, cisplatin, gemcitabine, and pegaspargase
mAb: Monoclonal antibody
ADC: Antibody-drug conjugate
BiTE: Bispecific T-cell engager
MM: Multiple myeloma
ADCC: Antibody dependent cytotoxicity
ADCP: Antibody dependent phagocytosis
CDC: Complement dependent cytotoxicity
ORR: Objective response rate
CR: Complete remission
PR: Partial remission
PFS: Progression-free survival
OS: Overall survival
PD-1: Programmed cell death protein 1
BV: Brentuximab vedotin
CHP: Cyclophosphamide, doxorubicin, and prednisone
PTCL: Peripheral T-cell lymphoma
MAD: Methotrexate, l-asparaginase, and dexamethasone
EPOCH: Etoposide, prednisone, vincristine, cyclophosphamide, and doxorubicin
IL-2R: Interleukin-2 receptor
CCR4: C-C chemokine receptor
CCL: CC motif ligand
TNFR: Tumor necrosis factor receptor
DLBCL: Diffuse large B-cell lymphoma
TCR: T cell receptor
ICI: Immune checkpoint inhibitors
PD-L1: Programmed cell death ligand 1
LMP1: Latent membrane protein 1
NF-κB: Nuclear factor κB
STAT3: Signal transducer and activator of transcription 3
CTL: Cytotoxic T lymphocyte
EBNA1: EBV nuclear antigen 1
HSCT: Hematopoietic stem cell transplantation
AspaMetDex: Pegaspargase, methotrexate, and dexamethasone
CTLA-4: Cytotoxic T lymphocyte-associated antigen 4
TIM-3: T-cell immunoglobulin-3
TIGIT: T-cell immunoglobulin and ITIM domain
BTLA: B/T lymphocyte attenuator
LAG-3: Lymphocyte-activation gene 3
CART: Chimeric antigen receptor T-cell
GEP: Genomic expression profiling
JAK/STAT: Janus-associated kinase/signal transducer and activator of transcription
VEGF: Vascular endothelial growth factor
PDGFR: Platelet-derived growth factor receptor
PI3K: Phosphatidylinositol 3-kinase
Akt: Protein kinase B
mTOR: Mammalian target of rapamycin
GIFOX: Gemcitabine, ifosfamide, oxaliplatin
XPO1: Exportin-1
DICE: Dexamethasone, ifosfamide, carboplatin, and etoposide
AURKA: Aurora kinase A
BCOR: BCL-6 corepressor
MLL2: Mixed lineage leukemia 2
HDACi: Histone deacetylase inhibitors
